# Acupuncture therapy for patients with hemifacial spasm: A protocol of systematic review and meta-analysis

**DOI:** 10.1097/MD.0000000000018329

**Published:** 2019-12-20

**Authors:** Han Yang, Jun Zhou, Dongling Zhong, Zihan Yin, Guixing Xu, Jiao Chen, Juan Li, Fanrong Liang

**Affiliations:** a College of Acupuncture and Moxibustion and Tuina; b College of Health Preservation and Rehabilitation, Chengdu University of Traditional Chinese Medicine, Chengdu, Sichuan, China.

**Keywords:** acupuncture therapy, effectiveness, hemifacial spasm, meta-analysis, protocol, safety, systematic review

## Abstract

**Background:**

Hemifacial spasm (HFS) brings a lot of trouble to patients’ daily life, having a severe influence on the psychological and physical wellbeing of patients. Relevant researches suggested that acupuncture therapy has potential benefits for HFS. However, there is no consistent conclusion. The purpose of our study is to assess whether acupuncture therapy is effective and safe for HFS.

**Methods:**

To collect relevant randomized controlled trials (RCTs), the following electronic databases will be searched: Web of Science, the Cochrane Library, EMBASE, MEDLINE, ISI Web of Knowledge, PsycINFO, Allied and Alternative Medieine, Chinese National Knowledge Infrastructure, Wanfang data, and Chinese Scientific Journals Database. We will take the cure rate and the total effective rate as the primary outcomes, and change in intensity after treatment, change in frequency after treatment, the recurrence rate, and adverse events as secondary outcomes. Endnote software 9.1 will be used for study selection, Review Manager software 5.3, and STATA 13.0 software will be used for analysis and synthesis.

**Results:**

Current relevant studies will be synthesized to assess whether acupuncture therapy is effective and safe for HFS.

**Conclusion:**

Our research will provide evidence of acupuncture therapy for HFS.

**Registration:**

International Prospective Register of Systematic Reviews (PROSPERO) CRD42019142473.

## 1 Introduction

Hemifacial spasm (HFS) is a neuromuscular disease that is featured by paroxysmal, involuntary twitching of unilateral, occasionally bilateral, facial muscles innervated by the facial nerve (seventh cranial nerve).^[1]^ It is a chronic progressive disease that usually occurs in one's 40s to 70s,^[2]^ but may also affect the adolescents,^[3]^ with higher prevalence in females (2:1).^[4]^ As reported in previous studies, the prevalence in Asians is relatively higher than that of whites.^[5,6]^ The common cause for HFS is compression on either the root exit of the facial nerve from the seventh brainstem or at its entering point to the auditory meatus.^[7–12]^ There is a significant influence of HFS on the psychological and physical well-being such as emotional disturbance, visual or speech disability.^[13–15]^ For instance, HFS might lead to impaired vision with drooping eyelids, which results in difficulties in reading and driving. Furthermore, involuntary facial spasm can evoke the risk of dysarthria.^[3,16]^ Also, it causes a lot of social awkwardness.^[16]^ HFS usually worsens gradually as the disease progresses, and is rarely self-limited,^[17]^ so it is necessary to treat in time.

The treatment of HFS mainly includes oral medications, botulinum neurotoxin injections, and surgical management.^[3]^ However, the currently existing treatment for HFS is unsatisfactory, due to limited effectiveness,^[16]^ undesirable side effects,^[18]^ the frequent repetition of procedures,^[19]^ considerable risks, and postoperative complications.^[20]^ Therefore, patients prefer seeking alternatives therapies for HFS.^[21]^

Acupuncture therapy, as a treasure of traditional Chinese medicine, has gained increasing attention worldwide. It has been used in a wide range of clinical situations and has achieved promising effectiveness. The advantages of acupuncture therapy in some other neurovascular conflict have been confirmed, such as trigeminal neuralgia,^[22,23]^ facial paralysis,^[24,25]^ and so on. There are studies suggesting that acupuncture therapy can be beneficial for HFS.^[26,27]^ Whereas the effectivenesses and safeties of acupuncture therapy for HFS remain unclear due to lack of comprehensive synthesis and evaluation of existing evidence. Hence, the purpose of our research is to systematically synthesize all randomized controlled trials (RCTs) of acupuncture therapy for HFS to provide evidence for the clinical practice of HFS.

## 2 Methods

### 2.1 Study registration

The protocol of our study has been registered on the International Prospective Register of Systematic Reviews (PROSPERO) (registration number, CRD42019142473). The protocol is reported strictly according to the Preferred Reporting Items for Systematic Reviews and Meta-Analyses Protocols (PRISMA-P) guidelines.

### 2.2 Eligibility criteria

#### 2.2.1 Type of study

We will include the RCTs of acupuncture therapy for patients with HFS.

#### 2.2.2 Type of participant

The study involved participants who had been diagnosed with HFS. Diagnostic criteria for HFS, according to “Neurology,^[28]^" “Clinical Neurosurgery,^[29]^" and “Clinical Pain Therapy^[30]^” include uncontrollable spasm on one side of the face, early manifestation as intermittent spasm of orbicularis oculi muscle, and gradually extends to mouth and finally affect the entire face; the severity of spasm varies and can be aggravated by stress, exhaustion, talking, and so on; spasm terminates when patients are asleep; also, there is no positive sign from neurologic examinations. There is no restriction of age, sex, or race.

#### 2.2.3 Type of intervention

Our research will include studies that took acupuncture therapy as the main treatment in the intervention group, such as acupuncture, auricular acupuncture, electroacupuncture, fire needle, scalp acupuncture, acupoint injection, press needle, acupressure, acupoint catgut embedding, among others. Meanwhile, the control group used nonacupuncture therapy (pharmacological treatments, conventional treatment, placebo, or waiting-list) or sham acupuncture.

#### 2.2.4 Types of outcome measurements

##### 2.2.4.1 Primary outcome

1. The cure rate (number of participants who made a full recovery/ total number of participants in this group);
2. The total effective rate (number of participants who showed a positive response to therapy/ total number of participants in this group).

##### 2.2.4.2 Secondary outcomes

1. Change in intensity after treatment;
2. Change in frequency after treatment;
3. The recurrence rate (number of people who relapsed during follow-up/ total number of participants in this group);
4. Adverse events related to interventions.

#### 2.2.5 Exclusion criteria

1. Participants with the unclear diagnosis;
2. Studies that did not use acupuncture therapy as primary treatment in the intervention group;
3. Data that cannot be extracted;
4. Duplicated data;
5. The studies where full text is unavailable.

### 2.3 Search methods for identification of studies

#### 2.3.1 Electronic data sources

The following 10 electronic databases from inception to November 2019 will be searched: Web of Science, the Cochrane Library, EMBASE, MEDLINE, ISI Web of Knowledge, PsycINFO, Allied and Alternative Medieine, Chinese National Knowledge Infrastructure, Wanfang data and Chinese Scientific Journals Database.

#### 2.3.2 Other resources

We will review and screen the relevant references. Furthermore, the following registration website of the clinical trial will be searched: WHO ICTRP, http://www.chictr.org.cn, http://www.ClinicalTrial.gov, and ISRCTN Register. In addition, we will search for the relevant gray literature from the Health Management Information Database, OpenSIGLE Database, and the National Technical Information Service. Experts in the field will be consulted for relevant studies.

### 2.4 Search strategy

We will combine subject words with text words for the search strategy. The search terms will be expanded around: acupuncture therapy, HFS, and randomized controlled trial. It will not be restricted with publication dates and languages. Use MEDLINE as an example, the specific searching strategy, as stated in Table 1. The searching strategy will be modified by the characteristic of the different databases.

**Table 1:**
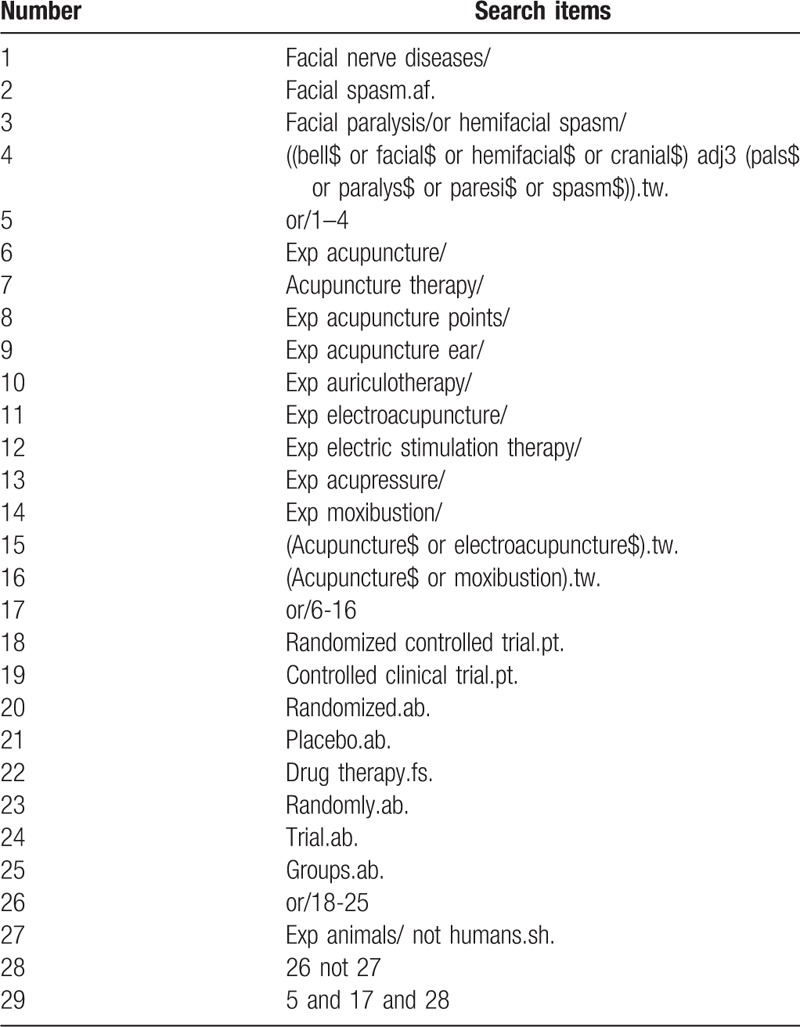
Search strategy for the MEDLINE database.

### 2.5 Data collection

#### 2.5.1 Selection of studies

**Figure 1:**
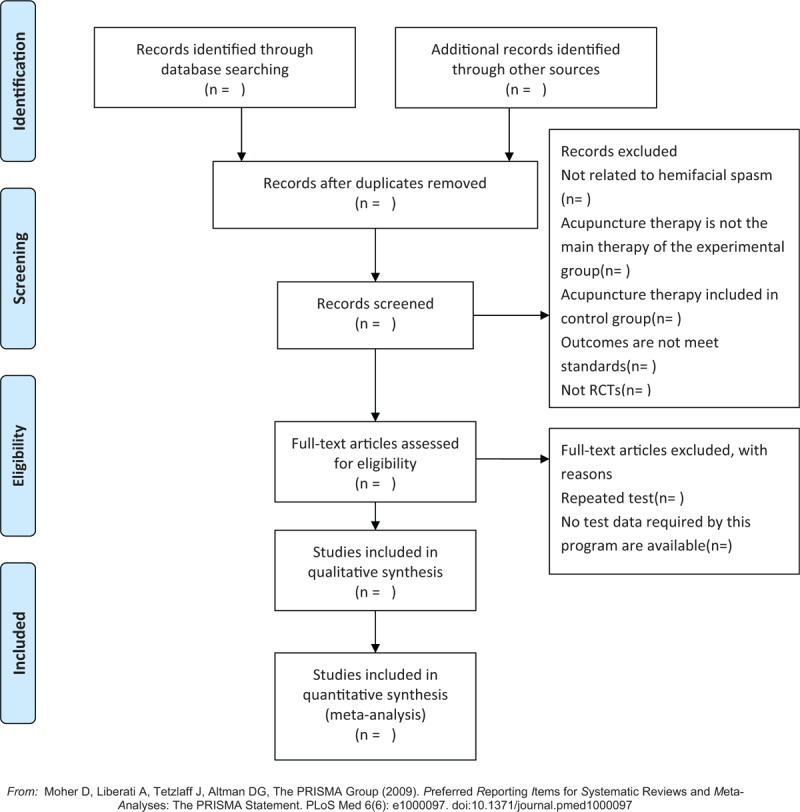
The Preferred Reporting Items for Systematic Reviews and Meta-analyses Protocols flow diagram of the study selection process.

We will import the retrieved studies in Endnote software 9.1 to remove duplicates. According to the established inclusion and exclusion criteria, 2 researchers (JZ and ZHY) will screen the titles and abstracts independently. After that, the full text will be screened as a second filtration. Two researchers will crosscheck the included studies, and the third researcher (FRL) will be involved if disagreements occur. The detailed screening process will be shown in the following PRISMA-P flow diagram (Fig. 1).

#### 2.5.2 Data extraction and management

The other 2 researchers (DLZ and GXX) will extract data independently to fill out the predesigned form. The information includes author, country, publication year, methodological quality, characteristics of participants, the details of intervention and comparisons, outcomes, the specific data, results, conclusions, follow-up, adverse events, conflicts of interest, sources of funds, and ethical approval. The extracted data will be cross-checked by the 2 researchers. A third researcher (FRL) will be involved if a disagreement occurs. The authors of the studies included will be contacted for further information when necessary.

#### 2.5.3 Assessment of risk of bias in included studies

According to the guidance from the Cochrane Handbook of Systematic Reviews of Interventions,^[31]^ 2 researchers (HY and JL) will evaluate the risk of bias of the included RCTs independently. We will evaluate from the following 6 parts: selection, performance, attrition, detection, reporting, and other sources of bias. We will rate the risk of bias into 3 levels: when meets none of the criteria, it will be regarded as high; when meets all criteria, it will be regarded as low; when study without sufficient information to determine, it will be regarded as unclear. After the assessment, it will be cross-checked by 2 researchers. The third researcher (FRL) will be involved if a disagreement occurs.

### 2.6 Data synthesis

Review Manager software (RevMan5.3) and STATA 13.0 software will be used to conduct all data analyses if it is possible to perform a meta-analysis. Data synthesis will be performed with a random-effects model if significant statistical heterogeneity is detected. Otherwise, the data will be processed with a fixed-effects model. Furthermore, the descriptive analysis will be conducted if there is significant statistical heterogeneity.

#### 2.6.1 Measures of treatment effect

For continuous outcomes (the change in intensity after treatment, the change in frequency after treatment), we will use mean difference to evaluate the extracted data. For dichotomous outcomes (the cure rate, the total effective rate, the recurrence rate, and adverse events), we will analyze the rate ratio. The confidence intervals (CIs) will be set to 95% for both continuous outcomes and dichotomous outcomes.

#### 2.6.2 Management of missing data

For the insufficient or missing data, the related corresponding author will be contacted. If we cannot get accurate data after contacting the corresponding author, these studies will be excluded.

#### 2.6.3 Assessment of heterogeneity

We will conduct the qualitative analysis by comparing the characteristics of included researches and quantitative analysis by using the *I*^2^ test and the *χ*^2^ test to assess the heterogeneity. If the values of *I*^2^ are >50%, the significant heterogeneity will be thought to exist.

#### 2.6.4 Assessment of reporting biases

When the quantity of the included RCTs ≥10, we will select funnel plots to evaluate the potential publication bias. Otherwise, we will use STATA 13.0 software to perform the Egger test.

#### 2.6.5 Subgroup analysis

According to different kinds of acupuncture therapy applied, different interventions of the control group, and different time points for evaluating outcomes after treatment, subgroup analysis will be performed.

#### 2.6.6 Sensitivity analysis

Based on the risk of bias, insufficient data, and sample size, we will perform a sensitivity analysis to evaluate the robustness if significant statistical heterogeneity existed.

### 2.7 Grading the quality of evidence

According to the Grading of Recommendations Assessment, Development, and Evaluation,^[32]^ we will assess the each outcome's quality of evidence from the 5 aspects (limitation of study design, inconsistency, indirectness, imprecision, and bias of publication) and rank the quality into 4 levels (very low, low, moderate, and high).

### 2.8 Ethics and dissemination

There is no necessity to gain ethical approval considering our research has no connection with individual patient data. The results of our research will be reported in a peer-reviewed journal or relevant conferences and evaluate the implication of acupuncture therapy for patients diagnosed with HFS.

## 3 Discussion

HFS brings a lot of trouble to patients’ daily life, having a severe influence on the psychological and physical well-being of patients. At present, the optimum treatment for HFS is still controversial. Acupuncture therapy may be an effective and safe treatment for HFS, which may work through regulating humoral immunity and auto cell immunity.^[33]^ However, there is no definitive conclusion. Following the Cochrane Handbook for Systematic Reviews of Interventions^[31]^ strictly, the systematic review and meta-analysis will be conducted based on the existing RCTs to assess whether acupuncture therapy is effective and safe for HFS, aiming to provide evidence for clinical practice and to facilitate future researches.

## Author contributions

The idea of this study was put forward by Han Yang and Dongling Zhong. The protocol was drafted by Han Yang and Jun Zhou. The whole process was supervised by Fanrong Liang. The manuscript was revised by all authors. The final version was approved by all authors.

**Conceptualization:** Han Yang, Dongling Zhong.

**Methodology:** Han Yang, Jun Zhou, Juan Li, Guixing Xu, Zihan Yin.

**Supervision:** Fanrong Liang.

**Writing – original draft:** Han Yang, Jiao Chen, Dongling Zhong.

**Writing – review & editing:** Han Yang, Jun Zhou, Juan Li.

## References

[1] Chopade TR Bollu PC Hemifacial Spasm. In: *StatPearls.* Treasure Island (FL): StatPearls Publishing; 2019. 30252364

[2] Czyz CN Burns JA Petrie TP Long-term botulinum toxin treatment of benign essential blepharospasm, hemifacial spasm, and Meige syndrome. Am J Ophthalmol 2013;156:173–7. 23541393 10.1016/j.ajo.2013.02.001

[3] Abbruzzese G Berardelli A Defazio G Hemifacial spasm. Handb Clin Neurol 2011;100:675–80. 21496615 10.1016/B978-0-444-52014-2.00048-3

[4] Auger RG Whisnant JP Hemifacial spasm in Rochester and Olmsted County, Minnesota, 1960 to 1984. Arch Neurol 1990;47:1233. 2241620 10.1001/archneur.1990.00530110095023

[5] Poungvarin N Devahastin V Viriyavejakul A Treatment of various movement disorders with botulinum A toxin injection: an experience of 900 patients. J Med Assoc Thai 1995;78:281–8. 7561552

[6] Wu Y Davidson AL Pan T Asian over-representation among patients with hemifacial spasm compared to patients with cranial–cervical dystonia. J Neurol Sci 2010;298:61–3. 20864122 10.1016/j.jns.2010.08.017

[7] Eckman PB Kramer RA Altrocchi PH Hemifacial spasm. Arch Neurol 1971;25:81–7. 5316997 10.1001/archneur.1971.00490010091012

[8] Tan EK Chan LL Clinico-radiologic correlation in unilateral and bilateral hemifacial spasm. J Neurol Sci 2004;222:59–64. 15240197 10.1016/j.jns.2004.04.004

[9] Jitpimolmard S Tiamkao S Laopaiboon M Long term results of botulinum toxin type A (Dysport) in the treatment of hemifacial spasm: a report of 175 cases. J Neurol Neurosurg Psychiatry 1998;64:751–7. 9647304 10.1136/jnnp.64.6.751 PMC2170133

[10] Iwakuma T Matsumoto A Nakamura N Hemifacial spasm. Comparison of three different operative procedures in 110 patients. J Neurosurg 1982;57:753. 7143057 10.3171/jns.1982.57.6.0753

[11] Jannetta PJ Abbasy M Maroon JC Etiology and definitive microsurgical treatment of hemifacial spasm. Operative techniques and results in 47 patients. J Neurosurg 1977;47:321–8. 894338 10.3171/jns.1977.47.3.0321

[12] Kenney C Jankovic J Botulinum toxin in the treatment of blepharospasm and hemifacial spasm. J Neural Transm 2008;115:585–91. 17558461 10.1007/s00702-007-0768-7

[13] Tan EK Fook-Chong S Lum SY Botulinum toxin improves quality of life in hemifacial spasm: validation of a questionnaire (HFS-30). J Neurol Sci 2004;219:151–5. 15050451 10.1016/j.jns.2004.01.010

[14] Rudzińska M Wójcik M Szczudlik A Hemifacial spasm non-motor and motor-related symptoms and their response to botulinum toxin therapy. J Neural Transm 2010;117:765–72. 20467763 10.1007/s00702-010-0416-5

[15] Tan EK Fook-Chong S Lum SY Case-control study of anxiety symptoms in hemifacial spasm. Mov Disord 2006;21:2145–9. 17044052 10.1002/mds.21150

[16] Wang A Jankovic J Hemifacial spasm: clinical findings and treatment. Muscle Nerve 1998;21:1740–7. 9843077 10.1002/(sici)1097-4598(199812)21:12<1740::aid-mus17>3.0.co;2-v

[17] JAM Jr Leone T Dhillon S Treatment choices of 119 patients with hemifacial spasm over 11 years. Clin Neurol Neurosurg 1996;98:213. 8884091 10.1016/0303-8467(96)00025-x

[18] Kemp LW Reich SG Hemifacial Spasm. Curr Treat Options Neurol 2004;6:175–9. 15043800 10.1007/s11940-004-0009-4

[19] Tan NC Chan LL Tan EK Hemifacial spasm and involuntary facial movements. QJM 2002;95:493. 12145388 10.1093/qjmed/95.8.493

[20] Mclaughlin MR Jannetta PJ Clyde BL Microvascular decompression of cranial nerves: lessons learned after 4400 operations. J Neurosurg 1999;90:1–8. 10.3171/jns.1999.90.1.0001 10413149

[21] Kilduff CL Casswell EJ Salam T Use of alleviating maneuvers for periocular facial dystonias. JAMA Ophthalmol 2016;134:1247. 27606483 10.1001/jamaophthalmol.2016.3277

[22] Gao J Zhao C Jiang W Effect of acupuncture on cognitive function and quality of life in patients with idiopathic trigeminal neuralgia. J Nerv Ment Dis 2019;207:171–4. 30720599 10.1097/NMD.0000000000000937

[23] Ichida MC Zemuner M Hosomi J Acupuncture treatment for idiopathic trigeminal neuralgia: a longitudinal case-control double blinded study. Chin J Integr Med 2017;23:829–36. 29080198 10.1007/s11655-017-2786-0

[24] Zhang R Wu T Wang R Compare the efficacy of acupuncture with drugs in the treatment of Bell's palsy: a systematic review and meta-analysis of RCTs. Medicine 2019;98:e15566. 31083225 10.1097/MD.0000000000015566 PMC6531040

[25] Kwon YJ Lee S Park S Clinical effects of needle-pricking therapy on peripheral facial paralysis. Forsch Komplementarmed 2014;21:14–8. 10.1159/000358742 24603625

[26] Zhang L Zhao L Bai Y Comparative observation of the efficacy on facial spasm among different therapies. Zhongguo Zhen Jiu 2017;37:35–8. 29231320 10.13703/j.0255-2930.2017.01.008

[27] Qian J Xu W Clinical observation of fire needle for facial spasm. Zhongguo Zhen Jiu 2015;35:1221–4. 26964161

[28] Jiang W Jianping J Liying C Neurology (Chinese). Beijing: People's Medical Publishing House; 2005.

[29] Chengyuan W Clinical neurosurgery (Chinese). Beijing: People's Medical Publishing House; 2007.

[30] Zhonglian L Jianxiong A Jiaxiang N Clinical Pain Therapy (Chinese). Tianjin: Tianjin Science & Technology Press; 1994.

[31] Higgins JP Altman DG Assessing Risk of Bias in Included Studies. 2011;Chichester, UK: John Wiley & Sons, Ltd, 187-241.

[32] Balshem H Helfand M Schünemann HJ GRADE guidelines: 3. Rating the quality of evidence. J Clin Epidemiol 2011;64:401–6. 21208779 10.1016/j.jclinepi.2010.07.015

[33] Yun X Kongjin G Effect of combined therapy with acupuncture and injectio ad acumen on T cell subset in peripheral blood of patients with hemifacial spasm. Journal o f Hainan Medical University 2011;17:330–2.

